# Exploring potential genes and pathways related to lung cancer: a graph theoretical analysis

**DOI:** 10.6026/97320630019954

**Published:** 2023-09-30

**Authors:** Shaheen Hayat, Romana Ishrat

**Affiliations:** 1 Centre for Interdisciplinary Research in Basic Sciences, Jamia Millia Islamia, Jamia Nagar, New Delhi-110025

**Keywords:** Differentially Expressed Genes, Protein-Protein Interaction Network, Lung Cancer

## Abstract

Lung cancer is the primary and third most frequently detected form of cancer in both males and females. The present study tries to perform integrated analysis in male as well as female patients inclusively both smoker and non-smokers. This study aims to identify diagnostic biomarkers and therapeutic targets for lung cancer patients using human microarray profile datasets. Differentially expressed genes (DEGs) were identified using a PPI network from the String database, and major modules or clusters were extracted using MCODE. The Cytohubba plug-in was used to find hub genes from the PPI network using centralities approaches. Twenty significant hub genes (CCND1, CDK1, CCNB1, CDH1, TP53, CTNNB1, EGFR, ESR1, CDK2, CCNA2, RHOA, EGF, FN1, HSP90AA1, STAT3, JUN, NOTCH1, IL6, SRC, and CD44) were identified as promising diagnostic biomarkers and therapeutic targets for lung cancer treatment. Survival analysis and hub gene validation were also conducted. GO enrichment and pathway analysis were conducted to identify their important functions. These hub genes were also used to identify targeted drugs. The findings suggest that the identified genes have the potential to be used as diagnostic biomarkers and therapeutic targets for lung cancer treatment.

## Background:

In the global context, lung cancer (LC) is identified as the primary and third most frequently detected form of cancer in males and females, respectively, in the year 2020. According to available data, it is anticipated that in the year 2020, there were approximately 2.2 million newly reported instances of LC globally. These cases accounted for 14.3% (1.4 million) and 8.4% (0.8 million) of all newly diagnosed cancer cases among males and females, respectively. The incidence rates of LC have exhibited a consistent downward trend among males in numerous high-income nations, such as those in Europe and Oceania, during the past few decades. Conversely, there has been a notable and rapid increase in LC incidence rates among females [1]. Cigarette smoking is widely recognised as the primary risk factor for lung cancer. Surprisingly, studies have shown that around 15% of male lung cancer patients and 53% of female patients who have been diagnosed with this disease have never smoked. Furthermore, research suggests that approximately 25% of lung cancer cases diagnosed globally cannot be directly linked to smoking [2, 3]. In addition, there has been an increase in lung cancer cases among non-smokers over time. This indicates that there is a distinct pathway for carcinogenesis, as well as differences in clinic pathological characteristics, epidemiology, and natural history compared to lung cancer in smokers [3, 4]. The increasing prevalence of cigarette smoking among women has resulted in a reduction of the disparity between male and female lung cancer incidence rates. However, it is evident that males consistently exhibit a higher number of cases and a relative disadvantage in terms of survival. Male individuals diagnosed with lung cancer exhibit a higher likelihood of mortality at the 5-year mark, regardless of factors such as stage, age, period of diagnosis, and histologic type [5, 6,7]. Lung cancer is characterised by genetic modifications such as mutations in the epidermal growth factor receptor (EGFR), fusion or rearrangement of the anaplastic lymphoma kinase (ALK), and various other abnormalities [8, 9]. Targeted therapy, such as the utilisation of tyrosine-kinase inhibitors, has the capacity to effectively limit the kinase activity of carcinogenic EGFR and ALK proteins [10]. There have been several studies conducted on signature genes identification and molecular mechanism in lung cancer, but they were only limited to particular sex (non-smoker females, smoker male) and individually smokers or non-smokers patients [11, 12,13,14]. Nevertheless, the precise molecular mechanisms underlying the tumorigenesis and progression of lung cancer in both sexes including smokers and non-smokers are still not fully understood. Hence, we tried to perform integrated analysis in male as well as female patients inclusively both smoker and non-smokers. In the present study, we have successfully discovered signature genes in lung cancer through the integration of bioinformatics analyses. The study focused on analysing the differentially expressed genes (DEGs) as well as the Gene enrichment and pathways that are linked to lung cancer. Following this, the validation of hub genes associated with lung cancer was performed. In addition, survival analyses were conducted to examine the correlation between the expressions of these genes. In this study, we analysed the proposed biomarkers to assess their potential use in the areas of diagnosis, prognosis, and performed drug-target interactions in lung cancer patients.

## Methodology:

## Inclusion Criteria for DEGs

A total of seven microarray datasets were manually retrieve from the NCBI GEO repository database for functional genomics data. It takes microarray & sequence-based data from gene profiling experiments [15]. These comprised of GSE151101, GSE151102, GSE151103 [16] GSE164750, GSE19804 [17, 18], GSE10072 [19] and GSE8987 [20] which were classified into normal and diseased, smokers and non-smokers groups. We made use of the GEO2R program for more exploration of the extracted microarray datasets. GEO2R is an interactive online program to find genes that are differentially expressed across experimental test conditions and users can compare & examine two or more samples in a GEO series using it [21]. GEO2R has in-built Limma R package [22]. In this analysis background correction and normalization were performed on the chosen lung cancer and normal control datasets using GEO2R. The outcomes were then downloaded in the MS Excel format. The datasets were prepared using the default parameters. Applying the parameters p < 0.05 and log fold-change |0.5-2| as the cut-off values, differentially expressed genes (up and down regulated genes) were retrieved. In this study, the log fold change |0.5-2| was used as a DEG screening condition.

## Gene ontology and Pathway Analysis of DEGs:

For the functional enrichment and pathway analyses of the differentially expressed genes in the present investigation, we used The Database for Annotation, Visualization, and Integrated Discovery (DAVID), which derives biological meaning from DEGs, lists [23]. Based on P-value, the top 10 entities from the KEGG pathways [24], biological process (BP), cellular component (CC), and molecular function (MF) categories were selected. Fisher's exact test is used by DAVID to enhance the functions of particular genes. P-values less than 0.05 were regarded as statistically significant.

## Construction of Protein-Protein Interaction Network:

In the PPI network, up-and down-regulated genes from meta-analyses were taken as genes of interest. By using String online database, the network was created. For a vast number of organisms with both functional and physical interactions, the STRING database gives anticipated and well-known protein-protein interactions data [25]. Further it provides associations and interactions not only from the combining experimental data already published and pathways from databases, but also from evolutionary relationships, ortholog based evidence, automatically curated biomedical literature and co-expression analysis. After that constructed network was imported into Cytoscape version 3.9.1 and visualization was done. The Cytoscape software gives more freedom to import additional data and it is developed for large number of network analysis and visualization [26]. To determine the crucial behaviours of the created PPI network, the following features were examined:

## Degree Distribution:

The degree (k) of a node (n) in a network research paradigm represents all connections or links with other nodes [27].

## Betweenness Centrality:

A measure of node's betweenness centrality in a network indicates how important it is for information to move from one node to another along the shortest possible routes [28].

## Closeness Centrality:

 The proximity centrality of a network measures how rapidly information is transmitted from one node to another [29].

## Stress:

Stress in a network is described as the sum of the nearby pathways of all node pairs [30].

## Module Finding From MCODE:

Molecular Complex Detection (MCODE) plugin was used in the Cytoscape to discover highly interconnected sections in the network for the cluster/module/community creation in the PPI network of genes. The plugin offers tools for analysing and observing protein interactions at the specific molecular level of interaction sites and modules. Along with "Degree cut-off = 2," "Node score cut-off = 0.2," "k-core = 2 (default more than 1)," and "max. Depth = 100," default statistical parameters were also employed. Protein modules involved in cellular processes are functional when they are bound to one another. Epochal modules can be recognised as highly interconnected subgraphs, and it is now unavoidable to use a variety of computational techniques to bring out them from complicated networks. Additionally, MCODE was used to the extremely compact modules to maintain the stability of the network [31, 32].

## Hub Gene Identification:

The process of identifying hub genes involved the utilisation of 12 algorithms for prediction within the cytohubba [33] a Cytoscape plug-in]. A set of the 20 best genes were identified as potential hub genes using every algorithm. Subsequently, only those genes that were identified by maximum of 6 algorithms were considered as hub genes.

## The Hub Genes Analysis in Lung Cancer:

GEPIA, also known as the Gene Expression Profiling Interactive Analysis, is a web server that allows users to analyse the expression profiles of tumours and normal samples. It utilises data from the TCGA (The Cancer Genome Atlas) and GTEx (Genotype-Tissue Expression) databases [34, 35]. The server can be accessed at [36], while the TCGA database can be found at [37] and the GTEx database at [38]. We used the pre-established criteria and set the cut-off value to 50%. The dataset used for analysis was the sample, and the hazard ratio (HR) was computed through the Cox proportional-hazards model. The present study did not calculate the 95% confidence interval (CI). In the field of HR, a p-value below the threshold of 0.05 was considered to be indicative of a statistically significant disparity. Furthermore, box plot analysis was conducted to validate the hub genes. Following that their clinical stage was determined.

## Drug-Gene Interactions:

The Drug-Gene Interaction Database (DGIdb) is a valuable resource for drug prediction. It allows users to screen drugs that have the potential to target specific genes of interest [39]. You can access the database at [40]. The DGIdb offers information on gene-drug interactions and the potential drug ability of genes based on their gene category. We used the DGIdb analysis to gather all potential gene-drug interactions for the hub genes.

## Results and Discussion:

## Gene Expression Profiling of Lung Cancer using Microarray Data:

This work compares analyses across datasets that are essential for comprehending gene activities in order to provide details about the structure of relationship among genes in various microarray datasets. According to Table 1, each series in the dataset has a unique number of differentially expressed genes. A comprehensive study of seven Gene Expression Omnibus (GEO) series resulted in the identification of a total of 2,369 differentially expressed genes (DEGs). Among these DEGs, 1,493 genes exhibited up-regulation, while 876 genes displayed down-regulation.

## Gene ontology and Pathway Analysis of DEGs:

The biological function and pathways enrichment were performed on DEGs. We discovered that the differentially expressed genes were notably enriched in various biological, cellular, and molecular processes, as well as in certain pathways. A simplified Fisher exact p-value of ≤0.05 is considered to indicate strong enrichment. The ten most enriched biological functions are listed in Table 2. Through our analysis of the biological processes (BP), we identified that the up-regulated genes are significantly enriched in several key processes. These processes include angiogenesis, extra cellular matrix organization, response to bacterium, and lung development. Additionally, down regulated genes were enriched in mitotic spindle assembly checkpoint, extra cellular matrix organization, mitotic spindle organization and chromosome segregation. Similarly, all the key processes and pathway enrichment details has been represented in following table.

## Protein-Protein Interaction Network Construction:

**Figure 1:**
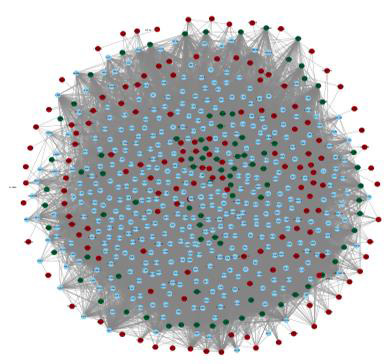
The Protein-Protein interaction Network. Nodes in the green colour represent downregulated genes, while red nodes show upregulated genes

**Figure 2:**
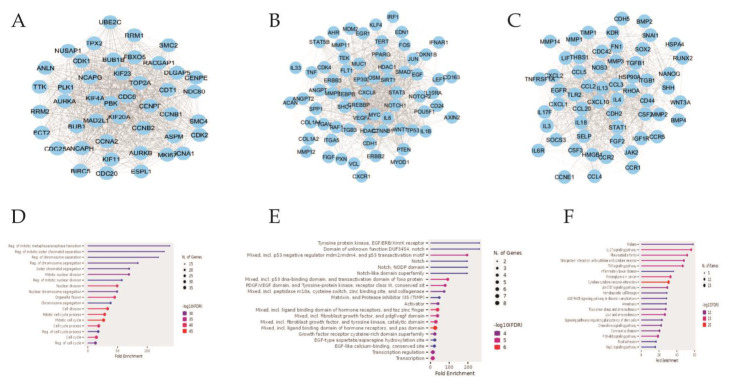
Three important modules and associated pathways A-C, three significant modules; D, pathways of module 1; E, pathways of module 2; F, pathways of module 3.

The resulting protein-protein interaction (PPI) network comprised of 472 nodes and 11,856 edges is illustrated in Figure 6. Subsequently, three essential modules or sub-networks were determined utilising the MCODE a Cytoscape plug-in. Furthermore, an analysis of the KEGG pathway enrichment was performed on identified modules (refer to Figure 2,Figure 1).

The genes of Module 1 exhibited enrichment in the reg. of mitotic metaphase/anaphase transition, mitotic sister chromatid separation, chromosome separation, as depicted in Figure 2D. Similarly, the genes of Module 2 demonstrated enrichment in tyrosine protein kinase, domain of unknown function DUF3454, notch, as shown in Figure 2E. Furthermore module 3 genes are enriched in malaria, IL-17 signalling pathway and rheumatoid arthritis (Figure 2F).

## Hub Genes Identification:

Twenty genes were identified as hub genes based on predictions from more than six cyto-Hubba algorithms. These genes include CCND1, CDK1, CCNB1, CDH1, TP53, CTNNB1, EGFR, ESR1, CDK2, CCNA2, RHOA, EGF, FN1, HSP90AA1, STAT3, JUN, NOTCH1, IL6, SRC, and CD44. Additionally, CCND1 was commonly predicted by nine different algorithms. The list of the top 20 hub genes, as identified by all 12 algorithms, can be found in: https://drive.google.com/drive/folders/1grA9vilvy-RM-JfhopknR4aQTSJ7m0c_?usp=sharing

## The Survival Analysis of Identified Hub Genes Using Gepia2:

**Figure 3:**
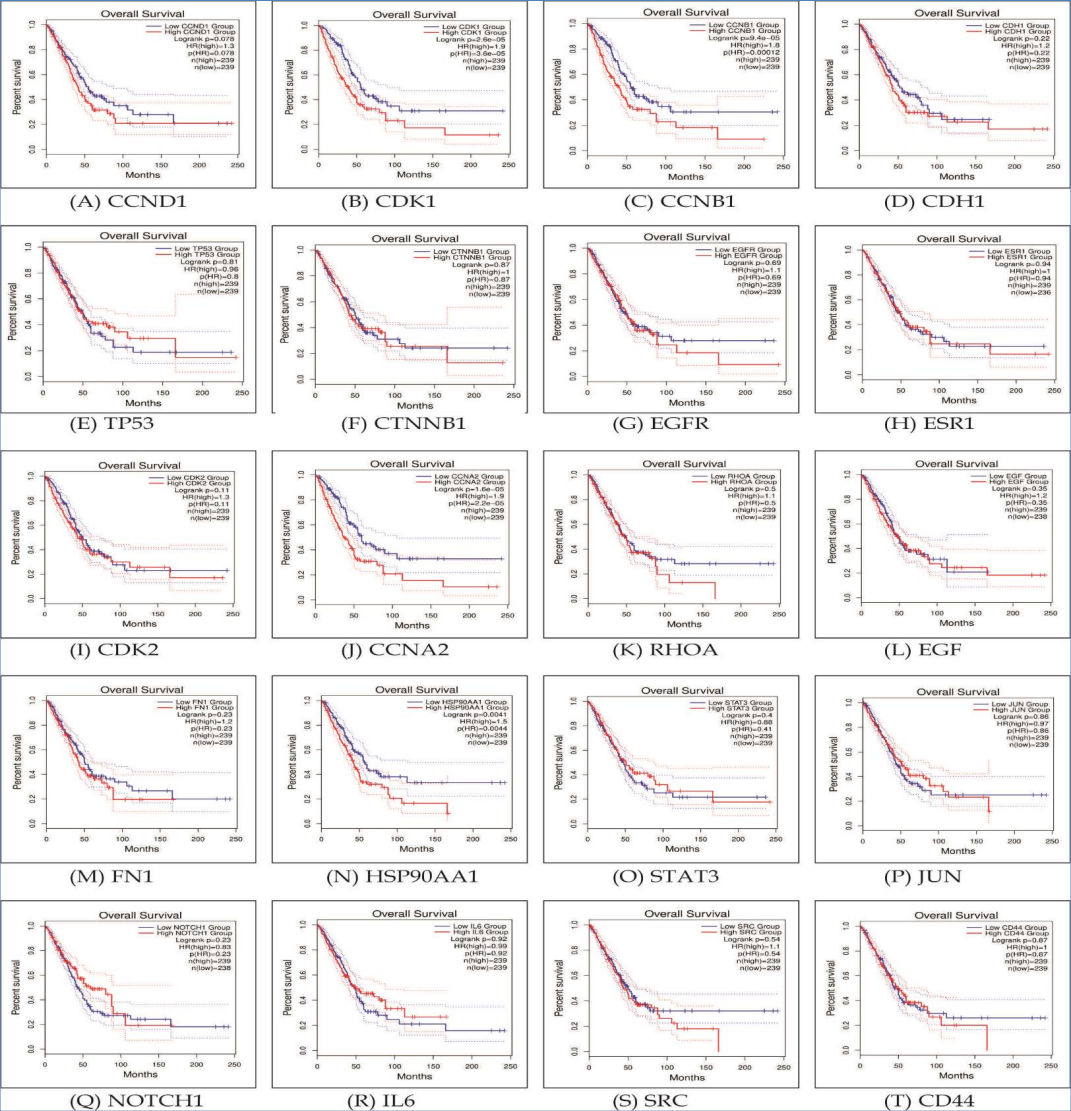
Survival curves of identified hub genes. (A-T) represents CCND1, CDK1, CCNB1, CDH1, TP53, CTNNB1, EGFR, ESR1, CDK2, CCNA2, RHOA, EGF, FN1, HSP90AA1, STAT3, JUN, NOTCH1, IL6, SRC, and CD44

We conducted a survival analysis on the chosen hub genes. Survival curves are utilized to demonstrate the ability to survive over time and the corresponding survival rate (Figure 3). The findings indicate that there is a significant association between high CDK1 gene expression (HR = 1.9) and poorer overall survival in patients with lung cancer. Similar associations were observed for CCNA2 (HR = 1.9), CCNB1 (HR = 1.8), HSP90AA1 (HR = 1.5), and IL6 (HR = 1.5).

**Figure 4:**
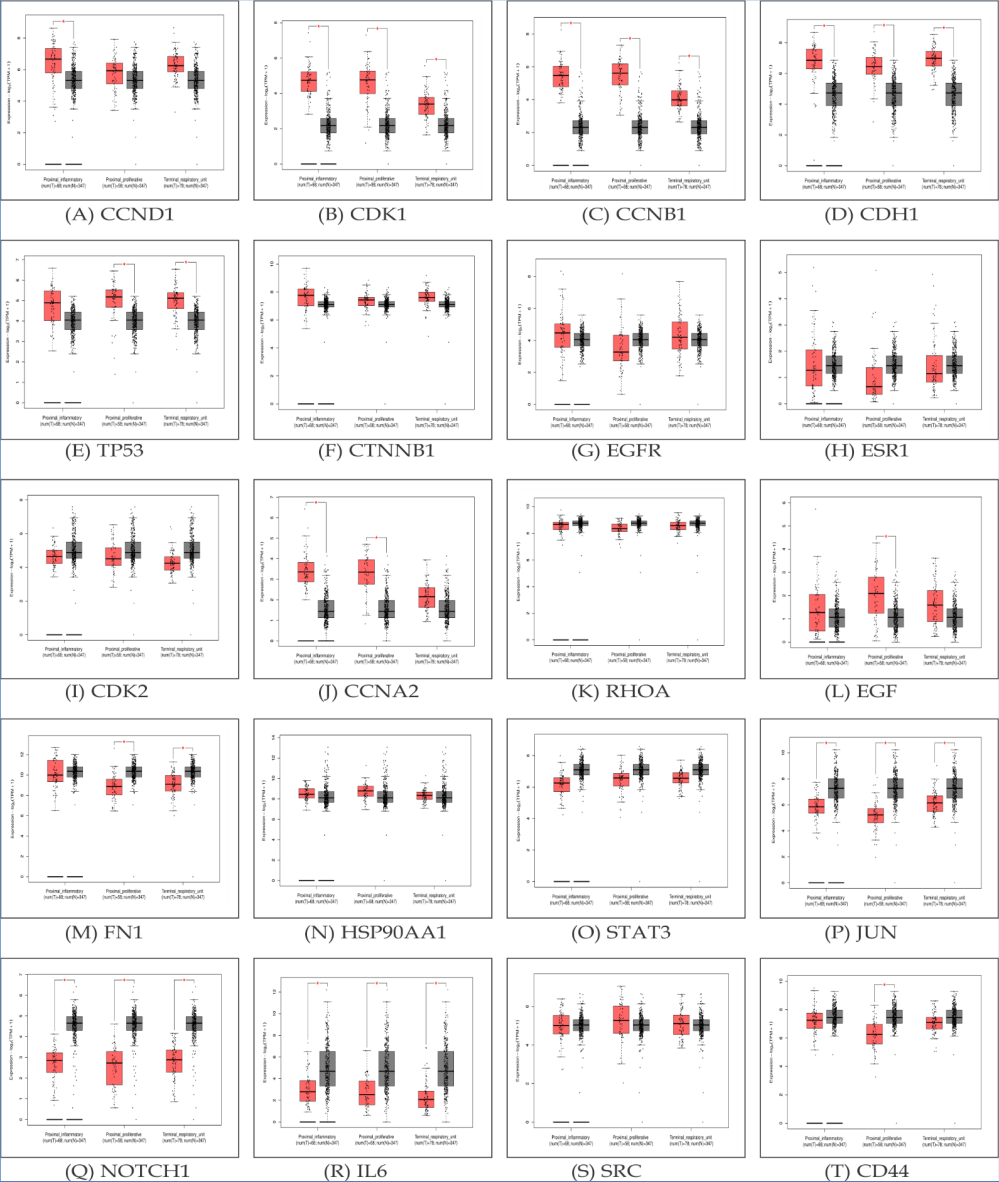
The box-plot representation of identified hub genes (A-T) based on TCGA database.

Additionally, we used the GEPIA tool for validating the hub genes expression through box plot analysis (Figure 4). The GEPIA box plots revealed that hub genes namely, CCND, CDK1, CCNB1, CDH1, TP53, CTNNB1, CCNA2, EGF, FN1, HSP90AA1, and SRC exhibited higher expression levels in lung tumor samples when compared to normal tissues.

**Figure 5:**
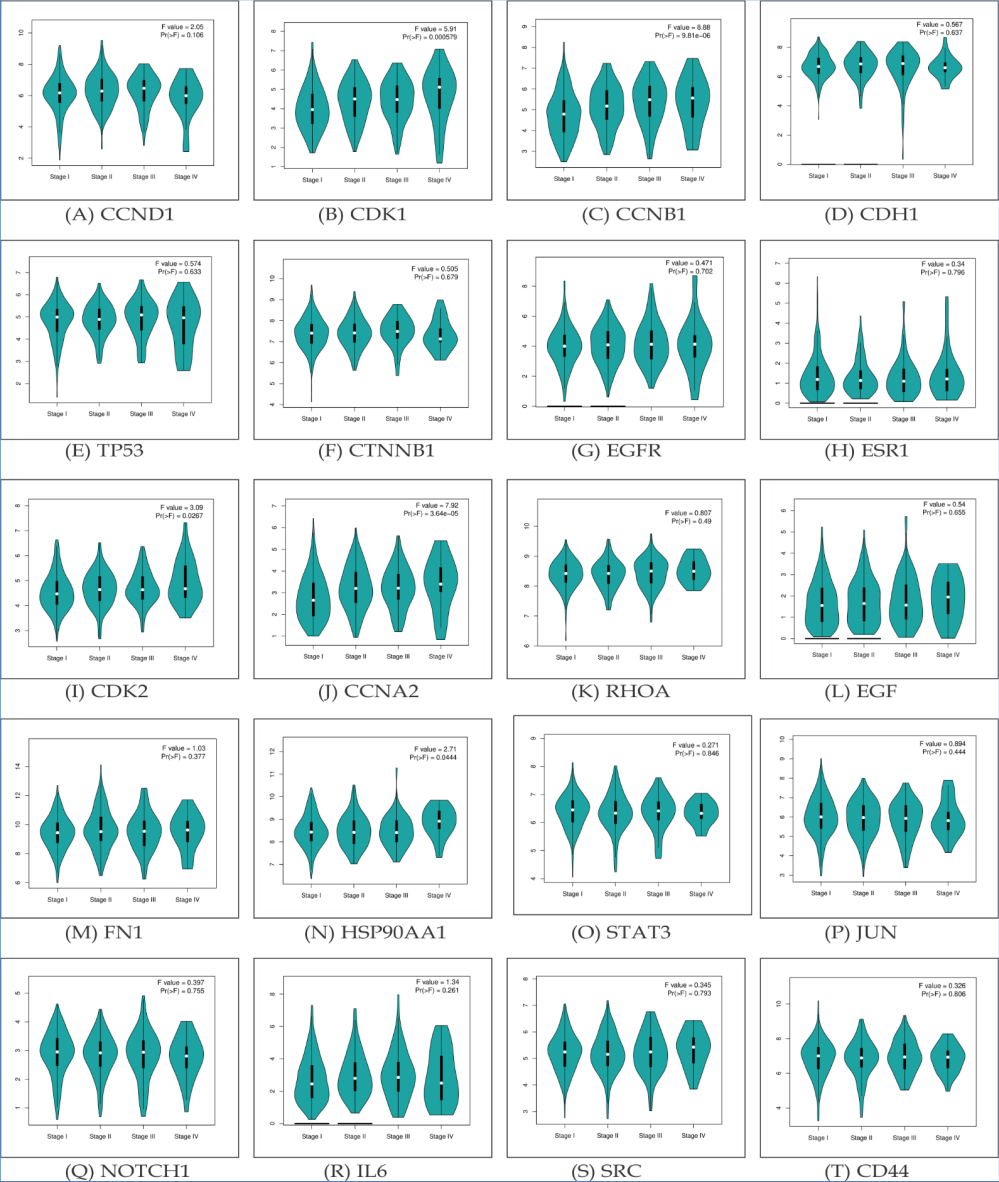
Representation of the correlation between hub gene expression and tumor stage in patients with lung cancer (A-T).

The relationship across hub gene expression and clinical stage in lung cancer patients was determined using a web server GEPIA (Figure 5). The violin plots illustrate the gene expression levels across pathological stages, as determined by the TCGA clinical annotation. The analysis demonstrates a significant association between high expression levels of these genes and advanced tumor stage.

## Drug-Gene Interactions:

**Figure 6:**
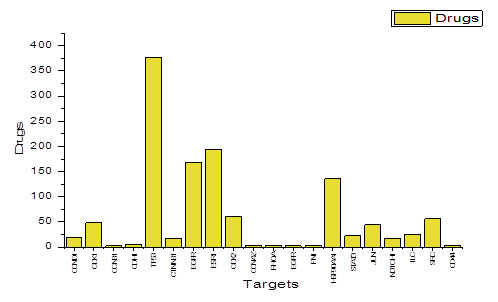
The Graph shows the number of interacting drugs with their key genes

The results indicate that all of the chosen hub genes identified their respective targets. A total of 1213 drugs were identified in DGIdb analysis. The figure 6 displays the total number of drugs that interact with specific target genes. The complete list of target genes and their associated drugs can be found in: https://drive.google.com/drive/folders/1grA9vilvy-RM-JfhopknR4aQTSJ7m0c_?usp=sharing

## Conclusion:

In summary, we conducted a bioinformatics analysis to identify key genes and pathways that are strongly associated with the development and progression of lung cancer in males and females including both smokers and non-smokers. This analysis focused on the differentially expressed genes (DEGs) found in cancerous lung tissues compared to normal lung tissues. The results offer valuable insights into the molecular mechanisms underlying the development of lung cancer. They also have the potential to identify diagnostic biomarkers and therapeutic targets. However, it is necessary to conduct additional molecular biological experiments in order to validate these findings.

## Figures and Tables

**Table 1:** Precise information of the datasets (microarray) associated with Lung Cancer.

**GEO Accession**	Platform	**Experiment Type**	**No. of Samples (controls/ disease)**	**Log Fold Change**	**DEGs (up/down)**
GSE151101	GPL11532	Expression Profiling by Array	237 Sample (109/128)	1.5	258/117
GSE151102	GPL11532	Expression Profiling by Array	123 Sample (59/64)	2	129/71
GSE151103	GPL11532	Expression Profiling by Array	360 Sample (172/188)	2	87/43
GSE164750	GPL570	Expression Profiling by Array	27 Sample (12/15)	2	643/462
GSE19804	GPL570	Expression Profiling by Array	120 Sample (60/60)	2	216/52
GSE10072	GPL96	Expression Profiling by Array	107 Sample (76/31)	0.5	83/54
GSE8987	GPL571	Expression Profiling by Array	15 Sample (7/8)	1.5	70/70

**Table 2:** The gene ontology and pathway enrichment of DEGs of Lung cancer

**Category**	**Term**	**Count**	**P-Value**	**Category**	**Term**	**Count**	**P-Value**
**Up Regulated DEGs**				**Down Regulated DEGs**			
	Angiogenesis	12	5.50E-06	BP	Mitotic Spindle Assembly Checkpoint	6	3.03E-07
BP	Extra cellular Matrix Organization	10	6.80E-06		Extra cellular Matrix Organization	7	0.00010139
	Receptor Internalization	6	4.65E-05		Mitotic Spindle Organization	5	0.000126594
	Response to Bacterium	8	5.07E-05		Chromosome Segregation	5	0.000385279
	Cell Adhesion	15	0.000137536		Collagen Catabolic Process	4	0.000832308
	BMP Signaling Pathway	6	0.000675601		Extra cellular Matrix Disassembly	4	0.001166144
	Lung Development	6	0.000786906		Inner Ear Development	4	0.001486291
	Cell-cell Adhesion	8	0.000810006		Apoptotic Process	10	0.001638216
	Calcium-mediated Signaling	6	0.000826883		Cell Division	8	0.001714427
	Complement Activation	4	0.000987328		Osteoblast Differentiation	5	0.002462495
CC	Extra cellular Region	46	3.18E-10	CC	Midbody	9	1.18E-06
	Extra cellular Space	38	2.51E-07		Spindle	7	3.93E-05
	Cell Surface	20	6.58E-07		Extra cellular Matrix	7	0.00082825
	Plasma Membrane	69	6.97E-07		Kinesin Complex	4	0.001392036
	Extra cellular Matrix	11	4.01E-05		Cornified Envelope	4	0.002004539
	External Side of Plasma Membrane	13	0.000259126		Extra cellular Space	18	0.002836245
	Integral Component of Membrane	62	0.000831799		Extra cellular Region	19	0.003133703
	Membrane Raft	9	0.000854365		Basolateral Plasma Membrane	6	0.003468987
	Receptor Complex	8	0.001635428		Kinetochore	5	0.004455837
	Integral Component of Plasma Membrane	23	0.003451787		Collagen Trimer	4	0.007006378
MF	Heparin Binding	10	1.24E-05	MF	Microtubule Binding	8	0.000197806
	G-protein Coupled Peptide Receptor Activity	4	0.005574687		Metalloendo Peptidase Activity	5	0.001992503
	Fibroblast Growth Factor Binding	3	0.014416929		Microtubule Motor Activity	4	0.002508748
	Interleukin-8 Receptor Activity	2	0.015941468		Protein Kinase Binding	8	0.008222867
	Transforming Growth Factor Beta Binding	3	0.016918063		ATP Binding	15	0.008850855
PATHWAY	Tryptophan Metabolism	4	0.007112033	PATHWAY	Cell Cycle	4	0.021901565
	Hypertrophic Cardiomyopathy	5	0.010137499		Bile Secretion	3	0.067149792
	Malaria	4	0.011507502		Signaling Pathway	3	0.073856423
	PPAR Signaling Pathway	4	0.033557595		Progesterone-Mediated Oocyte Maturation	3	0.085013868
	Tyrosine Metabolism	3	0.044998441				
	Complement and Coagulation Cascades	4	0.045941994				
	African Trypanosomiasis	3	0.0472853				
	Cell Adhesion Molecules	5	0.060546409				
	Dilated Cardiomyopathy	4	0.061814369				
	ABC Transporters	3	0.067006489				
